# Knowledge of non-communicable diseases and access to healthcare services among adults before and during COVID-19 pandemic in rural Tanzania

**DOI:** 10.3389/fpubh.2024.1342885

**Published:** 2024-03-28

**Authors:** Nathanael Sirili, Manase Kilonzi, George Kiwango, Edward Lengai, Ramla Nandala, Dorkasi L. Mwakawanga, Erick G. Philipo, Joseph Matobo Thobias, Gasto Frumence

**Affiliations:** ^1^School of Public Health and Social Sciences, Muhimbili University of Health and Allied Sciences, Dar es Salaam, Tanzania; ^2^School of Pharmacy, Muhimbili University of Health and Allied Sciences, Dar es Salaam, Tanzania; ^3^School of Medicine, Muhimbili University of Health and Allied Sciences, Dar es Salaam, Tanzania; ^4^Monduli District Council, Arusha, Tanzania; ^5^Kibaha District Council, Pwani Region, Tanzania; ^6^School of Nursing, Muhimbili University of Health and Allied Sciences, Dar es Salaam, Tanzania

**Keywords:** knowledge, access, healthcare services, COVID-19, rural, Tanzania

## Abstract

**Background:**

The COVID-19 pandemic significantly affected access to healthcare services, particularly among individuals living with Non-Communicable Diseases (NCDs) who require regular healthcare visits. Studies suggest that knowledge about a specific disease is closely linked to the ability to access services for that condition. In preparation for the future, we conducted the study to assess knowledge of NCDs and access to healthcare services among adults residing in rural areas before and during the COVID-19 pandemic.

**Methods:**

We conducted a community-based cross-sectional study in rural Tanzania in October 2022, a few months after the end of the third wave of the COVID-19 pandemic. A total of 689 community residents participated in the study. The level of knowledge of NCDs was assessed using an 11-item Likert questionnaire, which was later dichotomized into adequate and inadequate levels of knowledge. In addition, access to healthcare was assessed before and during the pandemic. We summarized the results using descriptive statistics and logistic regression was applied to determine factors associated with adequate levels of knowledge of NCDs. All statistical tests were two-sided; a *p*-value <0.05 was considered statistically significant. All data analyses were performed using SPSS.

**Results:**

Among 689 participants, more than half 369 (55%) had adequate knowledge of whether a disease is NCD or not; specifically, 495 (73.8%), 465 (69.3%), and 349 (52%) knew that hypertension, diabetes mellitus, and stroke are NCDs while 424 (63.2%) know that UTI is not NCD. Of the interviewed participants, 75 (11.2%) had at least one NCD. During the COVID-19 pandemic the majority 57 (72.2%) accessed healthcare services from nearby health facilities followed by traditional healers 10 (12.7%) and community drug outlets 8 (10.1%). Residence and education level were found to be significantly associated with knowledge of NCDs among participants.

**Conclusion:**

The study revealed that the community has a moderate level of knowledge of NCDs, and during the COVID-19 pandemic outbreaks, people living with NCD (s) relied on nearby health facilities to obtain healthcare services. Health system preparedness and response to pandemics should take into account empowering the community members to understand that NCD care is continuously needed even during pandemic times. We further advocate for a qualitative study to explore contextual factors influencing the knowledge of NCDs and access to healthcare services beyond the big domains of education and residence.

## Introduction

Non-communicable diseases (NCDs) are a significant health concern worldwide, responsible for about 70% of all deaths globally (1). Of all the deaths due to chronic diseases, 80% are from cardiovascular diseases and diabetes. In Africa, cardiovascular diseases are the second most common cause of death and are a significant contributor to the global burden (2, 3). Nevertheless, cancer and chronic obstructive pulmonary disease (COPD) also contribute significantly to the global burden of NCDs. It is estimated that by 2030, the global average age-standardized NCD mortality rate will be 510.54 per 100,000 population and NCDs will contribute to about 75% of total global deaths (4).

COVID-19 is a respiratory disease caused by coronavirus SARS-CoV-2. The disease emerged in the city of Wuhan, China, in December 2019 and is characterized by fever, dry cough, difficulty in breathing, or shortness of breath and symptoms of pneumonia. The disease quickly spread all over the world and in early 2020, COVID-19 was declared a global pandemic. During the COVID-19 outbreaks, global healthcare resources focused on viral management, the discovery of medicines, and prevention strategies like vaccine development (5). For instance, several clinical trials on drug repurposing of hydroxychloroquine and ivermectin alone or in combination for COVID-19 management were conducted while over 10 vaccines were formulated and approved for use worldwide (6). This resource reallocation disrupted the continuum of care for patients with NCDs (5). Also, during the pandemic, countries made tough decisions to safeguard their people. These decisions include lockdowns, social distancing, and mobilization of health personnel to the frontlines of the COVID-19 infection. The measures affected patients with NCDs who require regular hospital visits for follow-ups, check-ups, and prescription refills (5, 7, 8). Chudasama et al. conducted a global survey to assess the impact of the COVID-19 pandemic on routine care for chronic diseases among healthcare providers (HCPs) and reported that 67% rated moderate or severe effects on their patient management (5). In addition, 80% reported worsened mental health status of their patients (5). In South Africa, a qualitative study on the experience of patients with chronic diseases during the COVID-19 pandemic reported the following challenges; long waiting hours, limited number of patients seen per day, closure of health facilities, shortage of staff and medicines, negative staff’ attitude, and substandard chronic care management (9).

During the COVID-19 pandemic, NCD services in many countries were disrupted with a significant impact in low-income countries. More than half (53%) of the countries disrupted services for hypertension; 49% disrupted services for diabetes and diabetes-related complications; 42% disrupted cancer services, and 31% disrupted services for cardiovascular emergencies (10).

Coping with the pandemic while suffering from NCDs was a challenge and required someone to be knowledgeable of the diseases and thus access appropriate care (11). Literature supports that a community with good knowledge of NCDs will take appropriate preventive measures not to get the diseases and those with diseases will take appropriate measures to prevent poor progression (12). The latter has been proved through previous outbreaks in which community level of knowledge and perception were reported to affect activities implemented to prevent the spread of the disease (13–15). On the contrary, in many countries, people with adequate knowledge of NCDs is reported to be below 50%. In Asia, the proportion of the population with NCD knowledge ranged from 12.5% in Myanmar to 81.2% in Malaysia with China, Sri Lanka, and Saudi Arabia being below 50%. In Europe, a study from Spain revealed that 46.7% of the population had adequate knowledge of NCDs (16–22). Furthermore, health promotion and disease prevention about a certain disease is influenced by the social-demographic characteristics of the population including knowledge which in turn influences action to be taken (23).

Tanzania like other low-middle income countries (LMICs) was affected by the COVID-19 pandemic. According to the Tanzania Ministry of Health report of March 2021, 33,773 cases and 800 deaths were recorded (24). The most affected regions by the COVID-19 pandemic in Tanzania were Dar-es-salaam, Zanzibar, Pwani, Mwanza, Dodoma, Kagera, Manyara, and Morogoro (15). Measures executed to fight against transmission of COVID-19 in Tanzania were isolation, and care for infected people and suspected cases, observing physical distancing, the prohibition of mass gatherings, closure of schools and universities, community obliged to perform frequent handwashing, use of alcohol-based sanitizers, and wearing of face masks when move outsides homes (15). According to the WHO country disease outlook of 2023, NCDs contributed to 34% of the death records in 2019 in Tanzania and the leading NCDs include cardiovascular diseases, cancer, diabetes, and chronic respiratory disease (25). Despite literature supporting that people living with NCDs severely suffered during the COVID-19 pandemic few studies have documented how patients with NCD survived during the pandemic (26–28). Therefore, we conducted this study to assess knowledge of NCDs and access to healthcare services before and during the COVID-19 pandemic among the adult population in rural Tanzania.

## Methods

### Study design and setting

In October 2022, a community-based cross-sectional study was conducted to assess the knowledge of NCDs and access to healthcare services during COVID-19 among adults residing in rural and nomadic areas of Tanzania.

### Study population and eligibility criteria

The study population included community members aged 18 years and above living in the study area for at least 1 year.

### Sample size calculation

Using a formula for a single proportion by Kish and Lisle for an infinite population size, a minimum size of 640 participants was computed to answer the study objectives. Consider the following formula; *n* = z^2^* P(100-P) * Deff/ e^2^, whereby the proportion (p) of the population having adequate knowledge regarding COVID-19 prevention measures was assumed to be 50%, the margin of error of 5, 95% confidence interval and Deff of 1.5 (design effect). A minimum sample of 574.24 was obtained. We assumed a 10% non-response rate, and we obtained a minimum sample of 640. Provided two districts had two different population sizes, we used proportion sampling to select study participants from each district considering a ratio of 1:2.3 for Kibaha relative to Monduli. Therefore, a minimum of 194 participants were planned for Kibaha and 446 participants were planned for Monduli. The final sample size was 689, out of which 243 were from Kibaha and 446 from Monduli.

### Sampling strategy

The Multi-stage sampling strategy was adopted to recruit participants for this study as follows; In the first stage, we purposefully selected two regions of Arusha and Pwani from the Northern and eastern zones. Pwani is one of the regions that were badly hit by COVID-19 in the second and third waves while Arusha is among the regions that were not badly hit. Furthermore, the two regions were selected to represent a diversity of cultures from mostly nomads in Arusha to peasant petty traders in Pwani. In the second stage, from Pwani and Arusha, we randomly selected one district, Monduli district in Arusha and Kibaha district in Pwani. In the third stage, from each selected district, we listed all wards and stratified them into rural and urban wards, and from each stratum randomly selected one ward to be included in the study. From Monduli two wards were selected, Engutoto and Esilalei for urban and rural wards, respectively. Similarly, Mlandizi represents the urban ward and Kwala for the rural ward in the Kibaha district.

**Figure 1:**
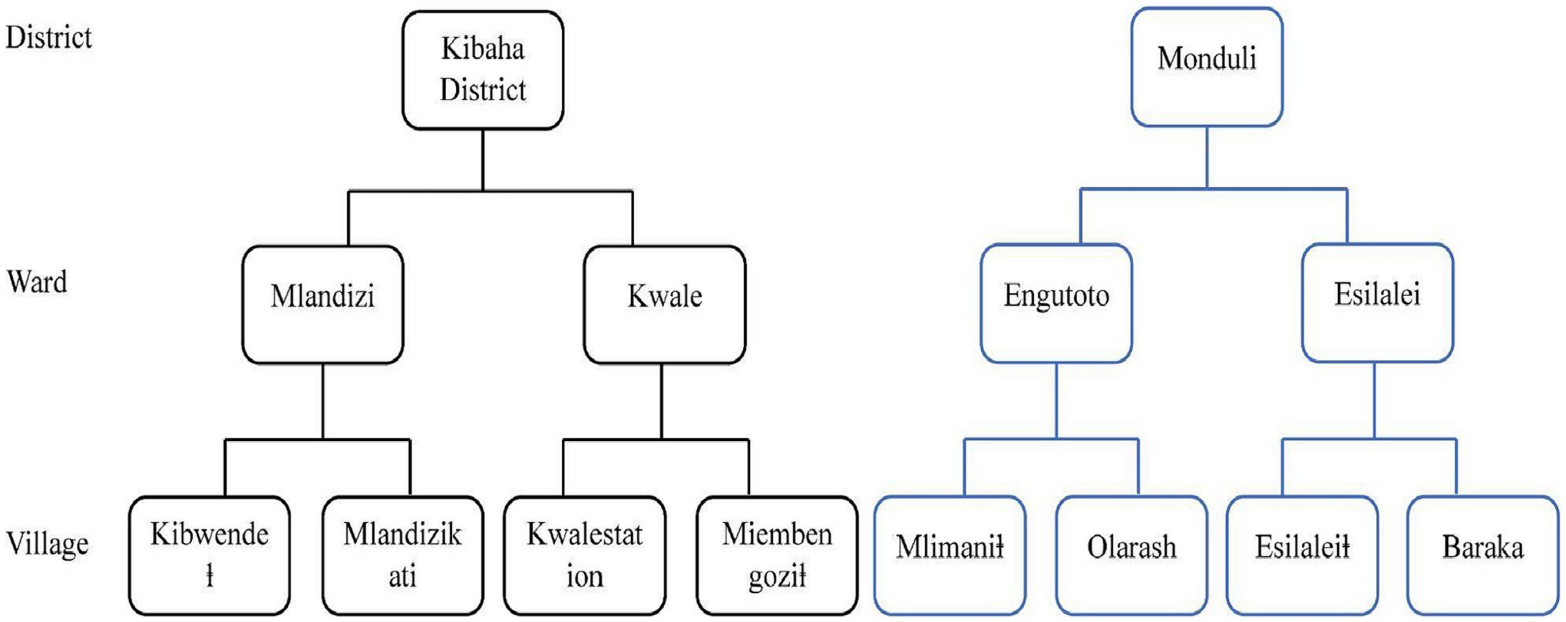
Flowchart of village election.

A ward is usually made up of several villages where community members live. Two villages were randomly selected from each ward, one with a healthcare facility and the other without a healthcare facility (Figure 1). In the fifth stage, we adopted a systematic sampling to include every nth household from the selected councils, wards, and villages. From each household, all adults aged 18 years and above were listed and grouped into men and women. Using a simple random sampling one man and one woman were interviewed. If a household had only one woman and a man, both were included. In households with only one individual, if aged above 18 years was included.

### Data collection procedure

A questionnaire with structured questions was prepared based on the literature review and the researchers’ knowledge of NCDs and access to healthcare services in developing countries. The questionnaire was developed in English and then translated into Swahili language. The questionnaire contained three main sections; section one collected demographic characteristics, section two contained a list of 11 diseases with the intention of assessing the knowledge of the participants on whether a disease is NCD or not, and the last section assessed access to healthcare services before and during COVID-19 pandemic.

In each district, the Community health system coordinator introduced the team of researchers to the wards and with the accompaniment of a ward official the team was introduced to the villages and hamlets. In each hamlet, a community health worker (CHW) introduced the research team to each household. Nine data collectors were recruited and trained on the purpose of the study, ethics, and the data collection tool. Four data collectors collected data in Kibaha and five in the Monduli district. The questionnaire was uploaded to Solstice software, a web-based data collection software, and tablets were used for data collection. One-to-one interviews were conducted with the selected household member and the data collector read the question and ticked the respective response.

All participants responded to questions on knowledge of NCD by responding to whether a disease is an NCD or not. Three -Likert scale questionnaires contained 11 diseases (a mixture of NCDs and communicable diseases) and the responses of either YES, NO or I DON'T KNOW were used. The correct response was rated 2, the wrong response 0 and I do not know 1. The mean was then used to determine whether a participant had adequate or inadequate knowledge of NCD diseases. We used the mean score as our measure of central tendency as the total scores of the participants were normally distributed after we performed the skewness test. Thereafter participants were asked whether they had any NCDs and the research assistants mentioned examples of NCDs and symptoms of NCDs to the participants. Those who said they have NCD were subjected to questions on access to healthcare services before and during COVID-19 outbreaks.

### Data analysis

Data from the Solstice software were retrieved into an MS Excel sheet and transferred to SPSS version 23 for analysis. Findings are summarized using frequency and percentages, and the median (interquartile range) is used to summarize age. The determinants of knowledge of the participants on whether a disease is NCD or not were screened using the Chi-square test and further (only those with a *p*-value of ≤0.2) were assessed using univariate and multivariate binary logistic regression analysis. A *p*-value of <0.05 was considered statistically significant.

### Institutional review board and informed consent statement

Ethical clearance for this study was obtained from the Muhimbili University of Health and Allied Sciences research and ethics committee with the registration number (MUHAS-REC-05-2022-1165). The permission to conduct data collection was obtained from the responsible ministry and respective district administration before data collection. Before the data collection process commenced, the study’s purpose was explained to the participants, and permission was granted. All methods were performed by the Declaration of Helsinki. All participants provided written informed consent before participating in the study.

## Results

### Demographic characteristics of the study participants

Out of the 689 recruited participants, 437 (63.4%) were female, and 189 (27.4%) were aged between (26–35) years. Most 513 (74.5%) of the participants were married, 541 (78.5%) were self-employed and 346 (50.2%) had primary education (Table 1).

**Table 1:** Demographic characteristics of the study participants (*n* = 689).

Variables	*n* (%)
Sex	
Male	252 (36.6)
Female	437 (63.4)
Median (Interquartile) age 35 (26–50) years	
Age (years)	
<25	168 (24.4)
26–35	189 (27.4)
36–45	126 (18.3)
46–55	82 (11.9)
56–65	65 (9.4)
>65	59 (8.6)
Marital status	
Married	513 (74.5)
Not married	176 (25.5)
Residence	
Mlandizi (urban ward)	104 (15.1)
Kwala (rural ward)	139 (20.2)
Engutoto (urban ward)	236 (34.3)
Esilalei (rural ward)	210 (30.5)
Employment status	
Employed	25 (3.6)
Not employed	123 (17.9)
Self-employed	541 (78.5)
Education level	
No formal education	190 (27.6)
Primary level	346 (50.2)
Secondary level	113 (16.4)
Tertiary level	40 (5.8)

### Knowledge of non-communicable among study participants

Overall, 369 (55%) participants have adequate knowledge of which disease is categorized as NCD or not. Specifically, 465 (69.3%) and 495 (73.8%) are aware that diabetes and hypertension are categorized as NCDs while 424 (63.2%) said that urinary tract infection (UTI) is not an NCD. Additionally, 333 (49.6%) said that malaria is an NCD while 375 (56.5%), 295 (44%), and 223 (33.2%) are not sure whether sickle cell disease, stroke, and epilepsy are categorized as NCDs, respectively, (Table 2).

**Table 2:** Knowledge of non-communicable diseases among study participants (*n* = 671).

Knowledge on whether a disease is categorized as NCD or not	*n* (%)
Diabetes	
Yes	465 (69.3)
No	42 (6.3)
I do not know	164 (24.4)
Hypertension	
Yes	495 (73.8)
No	24 (3.6)
I do not know	152 (22.7)
Urinary tract infections	
Yes	126 (18.8)
No	424 (63.2)
I do not know	121 (18)
Epilepsy	
Yes	352 (52.5)
No	96 (14.3)
I do not know	223 (33.2)
Cancer	
Yes	357 (53.2)
No	73 (10.9)
I do not know	241 (35.9)
Asthma	
Yes	281 (41.9)
No	150 (22.4)
I do not know	240 (35.8)
Stroke	
Yes	349 (52)
No	27 (4)
I do not know	295 (44)
Malaria	
Yes	333 (49.6)
No	235 (34.1)
I do not know	103 (15.4)
Heart diseases	
Yes	495 (73.8)
No	17 (2.5)
I do not know	159 (23.7)
Typhoid	
Yes	324 (47)
No	164 (24.4)
I do not know	183 (27.3)
Sickle cell disease	
Yes	261 (38.9)
No	31 (4.6)
I do not know	379 (56.5)
Overall knowledge of the participants on non-communicable diseases	
Adequate	369 (55)
Inadequate	302 (45)

### Access to healthcare services during COVID-19 among participants with NCD

Out of the 671 participants, 75 (11.2%) were having at least 1 NCD. Only 4 (5.3%) out of 75 were reported to have developed COVID-19 symptoms during the pandemic outbreak. The majority used to seek healthcare services from the nearby healthcare facilities 66 (81.5%), followed by traditional healers 9 (11.1%). During COVID-19 outbreaks the majority 57 (72.2%) sought healthcare services from nearby healthcare facilities followed by traditional healers 10 (12.7%), and community drug outlets 8 (10.1%) (Table 3). Also, 9 (12%) reported having faced treatment challenges during COVID-19 outbreaks. The reported challenges were lack of medications 5 (55.6%), shortage of healthcare providers 2 (22.2%), and shortages of other healthcare services 2 (22.2%).

**Table 3:** Place of access to healthcare services during COVID-19 among participants with NCD (*n* = 75).

Place of access	Period	
	Before COVID-19 pandemic (Response = 81)^*^	During COVID-19 pandemic (Response = 79)^*^
Health facility	66 (81.5)	57 (72.2)
Community drug outlets	4 (5)	8 (10.1)
Religious leaders/houses	2 (2.5)	2 (2.5)
Traditional healers	9 (11.1)	10 (12.7)
Others	0 (0.0)	2 (2.5)

^*^Some participants selected more than one places.

### Determinants of knowledge of whether a disease is categorized as NCD

Sex, age, marital status, employment status, and having NCD were found not to be associated with the level of knowledge of NCD among participants. However, on multivariate analysis, place of residence Engutoto [AR (95% CI) = 4.1 (2.34–7.24)] and Esilalei [AR (95% CI) = 2.5 (1.41–4.58)] and education level No formal education [AR (95% CI) =27.4 (7.62–98.48)], Primary education [AR (95% CI) = 8.5 (2.44–29.57)] and secondary education [AR (95% CI) =5.7 (1.59–20.71)] were significantly associated with level of knowledge of whether a disease is categorized as NCD or not (Table 4).

**Table 4:** Determinants of knowledge of whether a disease is categorized as NCD (*n* = 671).

Variables	Level of knowledge *n* (%)		*p*-value	COR (95% CI)	AOR (95% CI)
	Adequate	Inadequate			
Sex					
Male	133 (54.1)	113 (45.9)	0.17		
Female	236 (55.5)	189 (44.5)			
Age (years)					
<25	78 (47.9)	85 (52.1)			
26–35	105 (56.1)	82 (43.9)			
36–45	71 (57.7)	52 (42.3)	0.24		
46–55	51 (630)	30 (67)			
56–65	32 (50.8)	31 (49.2)			
>65	32 (59.3)	22 (40.7)			
Marital status					
Married	265 (53.3)	232 (46.7)	0.14	0.8 (0.54–1.09)	0.9 (0.63–1.38)
Not married	104 (59.8)	70 (40.2)		Ref	Ref
Residence					
Mlandizi (urban)	77 (75.5)	25 (24.5)		1.8 (1.01–3.04)	1.6 (0.95–2.84)
Engutoto (urban)	147 (63.1)	86 (36.9)	< 0.01	5.7 (3.33–9.71)	4.1 (2.34–7.24)
Esilalei (rural)	71 (35.1)	131 (64.9)		2.5 (1.42–4.40)	2.5 (1.41–4.58)
Kwala (rural)	74 (55.2)	60 (44.8)		Ref	Ref
Employment status					
Not employed	77 (63.1)	45 (36.9)		1.2 (0.50–3.11)	0.5 (0.18–1.39)
Self-employed	275 (52.5)	249 (47.5)	< 0.01	2 (0.82–4.54)	0.7 (0.26–1.79)
Employed	17 (68)	8 (32)		Ref	Ref
Education level					
No formal education	50 (28.2)	127 (71.8)		31 (9.24–106.24)	27.4 (7.62–98.48)
Primary level	203 (59.5)	138 (40.5)	< 0.01	8.4 (2.54–27.73)	8.5 (2.44–29.57)
Secondary level	79 (69.9)	34 (30.1)		5.3 (1.53–18.40)	5.7 (1.59–20.71)
Tertiary level	37 (92.5)	3 (7.5)		Ref	Ref
Have at least one NCD					
Yes	320 (53.7)	276 (46.3)	0.06	1.6 (0.98–2.79)	1.5 (0.85–2.56)
No	49 (65.3)	26 (34.7)		Ref	Ref

COR, Crude odds ratio; AOR, Adjusted odds ratio.

## Discussion

This study aimed to assess the level of knowledge of adult people residing in rural areas on NCDs and their access to healthcare services during COVID-19 outbreaks. The study found that 55% of the participants had adequate knowledge of NCDs; specifically, 73.8, 69.3, and 52% know that hypertension, diabetes mellitus, and stroke are NCDs while 63.2% know that UTI is not NCD. In addition, nearly half of the participants think malaria and typhoid are NCDs. Of the surveyed participants, 11.2% had at least one NCD and during the COVID-19 pandemic the majority obtained healthcare services from nearby health facilities (72.2%), traditional healers (12.7%), and community drug outlets (10.1%). In addition, lack of medications, and shortage of HCPs are the reported challenges encountered by the people with NCDs when seeking healthcare services during COVID-19 outbreaks. Moreover, residence and education level were found to be significantly associated with knowledge of NCDs among participants.

The level of knowledge of the community members observed in our study is comparable to other studies conducted in Saudi Arabia (43.8%), Sri –Lanka (43%), Spain (46.7%), and Rwanda (35%) (18–20, 29). The observed differences could be attributed to the study population, geographical location, and the types of questions used to assess knowledge of the NCDs. A study conducted in Bangladesh using a similar type of questions in assessing knowledge of NCDs among adults residing in rural settings found that 55.6, 56.3, and 42.1% know that diabetes, hypertension, and stroke are categorized as NCDs (30). The findings are low compared to our study; the inconsistency could be due to differences in the data collection period. Our study was conducted in 2022 soon after the end of the third wave while the Bangladesh study was conducted in 2018 before the COVID-19 pandemic outbreak (30). During the COVID-19 pandemic, awareness campaigns regarding the disease and prevention methods were established globally through platforms like television, radio, religious and political gatherings, social media, and newspapers (31). During the campaign people living with NCDs were identified as among the population being at high risk of COVID-19 morbidity and mortality (32). The observed raised in the level of knowledge of the community members on NCDs could be attributed to the campaigns during the COVID-19 pandemic.

The study demonstrated that the majority of community members are aware that urinary tract infections (UTIs) are not categorized as NCDs. Recently, UTI has been reported as one of the most common bacterial infections in low-middle-income countries (33), and because of the regular awareness campaigns on sexually transmitted infections (STIs) which often make a point to help individuals differentiate between the two diseases as they have similar pattern of symptoms (34). The latter could be a reason why the majority of the respondents in this study are aware of UTIs. In addition, despite Tanzania being one of the malaria-endemic regions, about half of the participants said that the disease is categorized as NCD. Recent reports on malaria indicate decreasing in prevalence in most of the endemic regions signifying that interventions are working (35). Nevertheless, the COVID-19 pandemic interrupted a lot of public health awareness intervention programs (26, 36). The finding serves as a warning to continue executing existing public health interventions regardless of the disease prevalence and during outbreaks.

Further, our study demonstrates that education level significantly determined the knowledge of the participants on NCDs. The findings are similar to several studies which reported that the level of education of participants determines their level of knowledge in different aspects (37, 38). Furthermore, those living in nomadic areas were significantly found to have inadequate knowledge. Literature supports that most of the nomadic people have poor education and poor access to different public health intervention campaigns (39). The findings suggest that alternative approaches should be developed to deliver information to hard-to-reach populations including nomads, and to ensure that they attend school.

Our study also observed a slight difference in the place where the majority of people reported having at least one NCD obtained healthcare services before and during COVID-19 outbreaks. A slight shift was observed from seeking healthcare services from the nearby health facilities to community drug outlets and traditional healers. The findings are supported by other studies which reported that during COVID-19 outbreaks the majority of patients were hesitant to visit health facilities due to fear of acquiring the disease (28, 40). Furthermore, people with NCD conditions like diabetes and cancer were told to be at high risk of getting severe complications of COVID-19 as well as high chances of death which affected their routine clinic attendance (41). Nevertheless, this study was conducted in rural areas in Tanzania, in which studies support that due to weak health systems and poverty in developing countries; community drug outlets and traditional healers serve as the primary point of acquiring healthcare services (42, 43).

Among the challenges in accessing healthcare services encountered by the people living with NCDs during the COVID-19 pandemic include a lack of medications and a shortage of HCPs in the healthcare facility. People with NCDs require medications all the time and require HCP intervention regularly, therefore, the reported shortages could be among the reasons why they severely suffered during the COVID-19 pandemic. The findings are supported by previous studies that reported interruption of medicines and medical devices supply chain in most countries during the COVID-19 pandemic (44, 45). The reallocation of healthcare providers to attend to COVID-19 patients has been associated with the reported shortage of providers in routine clinics during the outbreak.

Strengths and weaknesses of this study include the study design being cross-section fails to establish direct causal relationship between variables. Also, the study being quantitative fails to explore facts like how fear of getting COVID-19 affected health seeking behavior of the people living with NCDs. Furthermore, the sample size was calculated based on the objective of assessing community knowledge of NCDs, as a result, few participants responded to the questions regarding access to healthcare services before and during the COVID-19 pandemic. Moreover, in our study, we included study participants aged 18 years and above assuming that it is a population who can make freely informed consent despite understanding that there are individuals who are less than 18 years old and have NCDs. Nevertheless, the study recruited participants from different socioeconomic backgrounds, and the sample size is robust to make a conclusion.

## Conclusion

About half of the community has adequate knowledge of whether or not a disease is NCD. During the COVID-19 pandemic, the majority of participants obtained healthcare services from nearby healthcare facilities, while others relied on traditional healers and community drug outlets. Emphasis should be put on empowering the community with NCD knowledge and that continuity of NCD care is needed even during pandemic times. The latter requires joint efforts from the government and other health systems stakeholders responsible for strengthening health systems’ preparedness and responsiveness. In addition, qualitative studies are recommended to explore the reasons behind the reported poor health-seeking behavior among people living with NCD during the COVID-19 pandemic.

## Data availability statement

The raw data supporting the conclusions of this article will be made available by the authors, without undue reservation.

## Ethics statement

The studies involving humans were approved by Muhimbili University of Health and Allied Sciences research and ethics committee with the registration number (MUHAS-REC-05-2022-1165). The studies were conducted in accordance with the local legislation and institutional requirements. The participants provided their written informed consent to participate in this study.

## Author contributions

NS: Conceptualization, Project administration, Resources, Software, Supervision, Visualization, Writing – review & editing. MK: Data curation, Formal analysis, Methodology, Writing – original draft, Writing – review & editing. GK: Formal analysis, Methodology, Software, Supervision, Validation, Writing – review & editing. EL: Conceptualization, Project administration, Writing – review & editing. RN: Conceptualization, Project administration, Writing – review and editing. DM: Supervision, Validation, Visualization, Writing – review & editing. EP: Data curation, Formal analysis, Investigation, Software, Writing – review & editing. JT: Data curation, Formal analysis, Software, Validation, Visualization, Writing – review & editing. GF: Conceptualization, Funding acquisition, Methodology, Project administration, Resources, Supervision, Writing – review & editing.
